# Structures of Mature and Urea-Treated Empty Bacteriophage T5: Insights into Siphophage Infection and DNA Ejection

**DOI:** 10.3390/ijms25158479

**Published:** 2024-08-03

**Authors:** Yuning Peng, Huanrong Tang, Hao Xiao, Wenyuan Chen, Jingdong Song, Jing Zheng, Hongrong Liu

**Affiliations:** 1 Institute of Interdisciplinary Studies, Key Laboratory for Matter Microstructure and Function of Hunan Province, Key Laboratory of Low-Dimensional Quantum Structures and Quantum Control, School of Physics and Electronics, Hunan Normal University, Changsha 410082, China; ynpeng0808@163.com (Y.P.); xiaohao201709@163.com (H.X.); wenyuanchen@hunnu.edu.cn (W.C.); 2 National Key Laboratory of Intelligent Tracking and Forecasting for Infectious Diseases, National Institute for Viral Disease Control and Prevention, Chinese Center for Disease Control and Prevention, Beijing 100052, China; songjd@ivdc.chinacdc.cn; 3 School of Computer Science, Xiangtan University, Xiangtan 411105, China; tanghuanrong@126.com

**Keywords:** bacteriophage T5, siphophage, urea-treated empty particle, connector complex, cryo-EM

## Abstract

T5 is a siphophage that has been extensively studied by structural and biochemical methods. However, the complete in situ structures of T5 before and after DNA ejection remain unknown. In this study, we used cryo-electron microscopy (cryo-EM) to determine the structures of mature T5 (a laboratory-adapted, fiberless T5 mutant) and urea-treated empty T5 (lacking the tip complex) at near-atomic resolutions. Atomic models of the head, connector complex, tail tube, and tail tip were built for mature T5, and atomic models of the connector complex, comprising the portal protein pb7, adaptor protein p144, and tail terminator protein p142, were built for urea-treated empty T5. Our findings revealed that the aforementioned proteins did not undergo global conformational changes before and after DNA ejection, indicating that these structural features were conserved among most myophages and siphophages. The present study elucidates the underlying mechanisms of siphophage infection and DNA ejection.

## 1. Introduction

Siphophages have a long, flexible, and non-contractile tail that is connected to the head via a connector complex [1,2]. The tail, which comprises a tail tube, a tape measure protein (TMP) complex, a tail tip complex (or baseplate), and several side fibers [3], plays a pivotal role in host recognition, cell wall perforation, and DNA ejection. The connector complex of siphophages typically comprises a portal, an adaptor, a stopper, and a tail terminator. This complex exhibits structural similarities with that of myophages [4]. Although the mechanisms of the infection and DNA ejection of podophages [5,6,7,8] and myophages [9,10,11,12] have been extensively studied by cryo-EM single particle and tomography, similar research for siphophages is a challenge because of their highly flexible tail. Researchers have determined the medium- to high-resolution structures of several monomeric proteins and isolated complexes, such as the connector complex [13], the tail tube protein (TTP) [14,15,16,17], and the tail tip complex [18,19,20], and the medium-resolution structures of some intact siphophages have been obtained [3,21,22]. However, very few intact siphophages, such as Lambda [23], DT57C [24], and R4C [25], have been resolved to near-atomic resolutions. In addition, to the best of our knowledge, no intact high-resolution structure of DNA-ejected siphophages has been reported to date. Although the mechanisms of siphophage infection and DNA ejection have been elucidated [1,2], the precise conformational changes in protein components remain unclear for most siphophages due to a lack of high-resolution structures for DNA-ejected siphophages.

Bacteriophage T5, a representative member of the T5-like family of siphophages, serves as an excellent model for studying the assembly, infection, cell wall perforation, and DNA ejection mechanisms of this phage family. Comprehensive structural [14,18,26] and biochemical [27,28,29] studies of T5 have provided valuable insights into these mechanisms. The head of T5 comprises major capsid protein (MCP) pb8 and decoration protein pb10 [26]. The tail comprises TTP pb6 [14], TMP pb2, and a tail tip complex [18]. The tail tip complex comprises multiple protein components, including p140, collar protein p132, distal tail protein pb9, baseplate hub proteins pb3 and p143, central fiber protein pb4, and receptor-binding protein (RBP) pb5, all of which are surrounded by the side tail fibers pb1. T5 initiates infection by reversibly binding to the host cell’s lipopolysaccharide (LPS) O-antigen through fibers pb1 [30]. The RBP pb5 binds to its receptor, FhuA [31], which induces a series of substantial conformational changes. These include the bending of pb4 at the side of the tail tip, binding the tail to the membrane, the expulsion of TMP, the opening of TTP, and the formation of a transmembrane channel [18]. Notably, TTP does not directly contribute to infection or DNA ejection [14]. While the structures of T5′s isolated TTP [14] and tip complex [18] have been elucidated in both pre- and post-DNA ejection states, the integration of these components into a complete structure remains to be undertaken. Moreover, the structure of the T5 connector complex in the aforementioned two states must be determined to comprehend the function of this complex before and after DNA ejection.

The siphophage DT57C is a member of the T5-like family [24]. Despite sharing structural and sequence-related similarities, e.g., in the MCP, connector, and TTP, DT57C and T5 exhibit notable differences in the decoration protein, TMP, side tail fiber, and RBP. These differences may be of crucial importance in the interactions of DT57C and T5 with host cells. Moreover, the structure of the DNA-ejected DT57C has not yet been resolved to a near-atomic resolution. In this study, we obtained a laboratory-adapted, fiberless T5 mutant [28] and resolved the in situ structures of mature T5 and urea-treated empty T5 at near-atomic resolutions using cryo-EM. These structures enabled us to build more accurate atomic models for the MCP pb8 and decoration protein pb10. Furthermore, we resolved the structures of the connector complex, comprising portal protein pb7, adaptor protein p144, and tail terminator protein p142, were resolved in two states. Additionally, the structure of the tip complex, lacking side tail fibers pb1 and collar protein p132, was resolved in mature T5. A structural comparison of the connector complex between the two states revealed that the connector did not undergo any global conformational change. On the basis of a previously reported tip complex model [18] and our new structures, we present a complete DNA-ejected T5 model, which provides insights into the mechanisms of siphophage infection and DNA ejection.

## 2. Results

### 2.1. Overall Structures of Mature T5 and Urea-Treated Empty T5

Mature T5 was purified from *Escherichia coli* for cryo-EM data collection (Figure S1A), and empty T5 was obtained by treating mature T5 with urea (Figure S1B). Totals of 44,691 mature particles and 76,271 urea-treated empty particles were extracted from 3310 and 3897 cryo-EM micrographs, respectively. We obtained a high-resolution head map of mature T5 at a resolution of 3.9 Å by using icosahedral reconstruction. Using the local reconstruction method [32,33], we further improved the three-fold region of the head to a resolution of 3.4 Å (Figure S1A). Subsequently, we obtained an asymmetric head-connector structure of mature T5 at a resolution of 10.5 Å by using the symmetry-mismatch reconstruction method [33,34] (Figure S1A). Using the local reconstruction method [32,33], we then obtained the structures of the connector complex and the portal of mature T5 at resolutions of 4.8 and 3.2 Å (Figure S1A), respectively. We manually selected the tail tube and tail tip complexes from the micrographs by using the RELION 3.0 software [35] and finally obtained the structures of the tail tube and tail tip complex at resolutions of 3.6 and 3.9 Å (Figure S1A), respectively. In contrast to previous studies on mature T5 [14,18,26], the new density maps allowed us to build more accurate atomic models for MCP pb8 and decoration protein pb10 (Figure S2). Furthermore, we built atomic models for the N-terminus of TTP pb6 and the tip complex, including distal tail protein pb9, p140 and baseplate hub protein pb3, as well as for the C-terminus of TMP pb2. These models were consistent with those in previous T5 structures [14,18]. However, side tail fiber pb1 and collar protein p132 could not be detected (Figure S3). We resolved the structure of the connector complex, comprising portal protein pb7, adaptor protein p144, and tail terminator protein p142 (Figure S3). However, we were unable to resolve central fiber protein pb4, RBP pb5, the N-terminus of TMP pb2, or the C-terminus of TTP pb6 to sufficiently high resolutions, possibly due to their flexibility. Resolution was estimated using the Fourier shell correlation = 0.143 criterion, in accordance with the “gold standard” method [36]. The data acquisition and reconstruction statistics are presented in Table S1.

**Figure 1:**
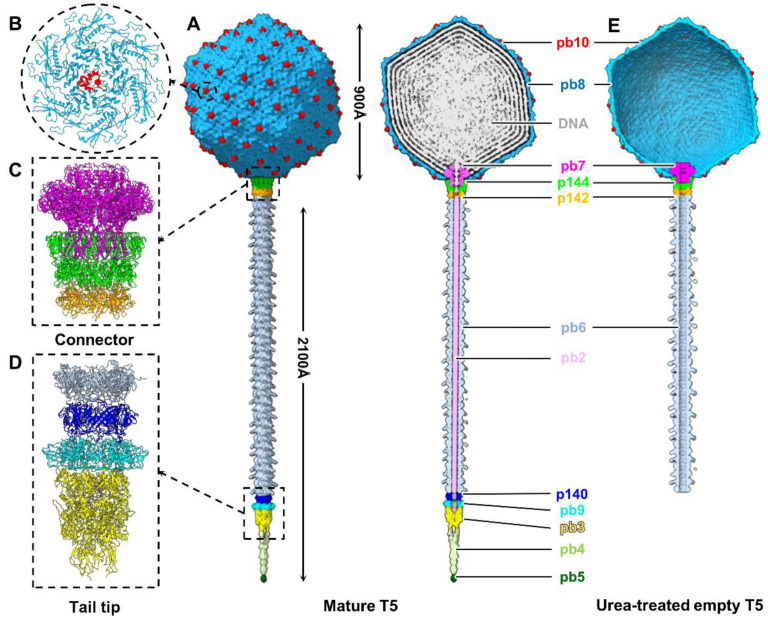
Overall structures of mature and urea-treated empty T5. (**A**) Side (left) and cut-open (right) views of the entire asymmetric structure of mature T5. The color code applies to panels A-E. (**B**) Zoomed-in view of the interactions between the MCP pb8 and decoration protein pb10. (**C**,**D**) Side views of ribbon models of the connector (**C**) and tip (**D**) complexes. (**E**) Cut-open view of the asymmetric structure of urea-treated empty T5 lacking the tail tip complex.

The T5 head comprises 775 copies of MCP gp8, arranged into 11 pentons and 120 hexons, forming an icosahedral shell with a triangle number (T) of 13 (Figure 1A,B). The center of each hexon is decorated with decoration protein pb10 (Figure 1B). The T5 tail, which comprises 40 trimeric rings of the TTP and a distal tip complex, is connected to a unique five-fold vertex of the head by the connector complex. The channel of the tail tube is occupied by TMP pb2 (Figure 1A). The connector complex comprises two dodecameric portal protein pb7 and adaptor protein p144, and a hexameric tail terminator protein p142 (Figure 1C). The tip complex contains trimeric p140, 6-fold distal tail protein pb9, and trimeric hub protein pb3 (Figure 1D).

Using the aforementioned method, we obtained an asymmetric head-connector structure of urea-treated T5 at a resolution of 7.5 Å and the structure of its connector complex at a resolution of 4.2 Å (Figure S1B). We built atomic models for the portal, adaptor, and tail terminator of urea-treated empty T5 (Figure S4). In contrast to the mature T5, the DNA and TMP are ejected in urea-treated empty T5, resulting in an empty head and an unoccupied tail tube (Figure 1E).

### 2.2. Structure of the Head

**Figure 2:**
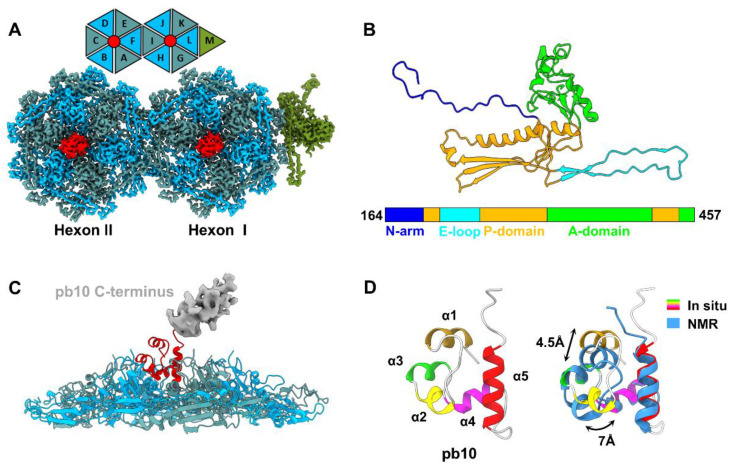
Structures of the MCP and decoration protein of mature T5. (**A**) Cartoon and density maps showing the 13 MCP monomers (labeled A–M) and two decoration protein monomers in an asymmetric unit of the icosahedral head. The 12 monomers from 2 types of hexons are colored cadet blue and sky blue; the 13th monomer from a penton is colored green; and the two decoration proteins are colored red. (**B**) Ribbon model of MCP pb8. (**C**) Interactions between a hexon and decoration protein pb10. The color coding is identical to that of Figure 2A, except for the flexible C-terminus (gray) of pb10. (**D**) Left: Ribbon model of the N-terminus of pb10. Right: Structural comparison between our in situ model and NMR model of pb10 (PDB ID: 5LXL), revealing slight conformational changes.

In comparison with the previous study of the T5 head [26], we built a more accurate atomic model of the pb8 monomer from residues 164–457 (out of 458 residues) based on our high-resolution density map (Figure 2A and Figure S5). MCP pb8 adopts a canonical HK97-like fold [37] and can be divided into four domains (Figure 2B and Figure S5): an extended N-arm (residues 164–191), a P-domain (residues 192–205, 245–306, and 407–451), an E-loop domain (residues 206–244), and a complex A-domain (residues 307–406, and 452–457). During the maturation of the capsid, the first 160 residues of the N-terminus of pb8 undergo proteolytic cleavage [26], and this process also occurs in the MCPs of E217 [10] and HK97 [37]. The asymmetric unit of the icosahedral head comprises 13 MCP gp8 monomers (a fifth penton, a hexon I, and a hexon II) and 2 pb10 monomers (Figure 2A). The center of each hexon is adorned with a decorating protein pb10, which comprises an α-helical domain and an immunoglobulin (Ig)-like domain (Figure 2C) [38]. We resolved only the α-helical domain (Figure 2D), from residues 2 to 68 (out of 164 residues), which contains five α-helices: α1 (residues 4–10), α2 (residues 17–24), α3 (residues 25–30), α4 (residues 33–41), and α5 (residues 49–62). A structural comparison of our α-helical domain with the nuclear magnetic resonance (NMR) structure of pb10 in solution [38] reveals that the α1 and α2 of the pb10 monomer undergo substantial conformational changes from the solution state to the assembled state (Figure 2D). All gp10 proteins bind to hexons with notably high affinities, primarily through a series of hydrogen bonds and salt bridges (Figure S6). Notably, pb10 is proposed to enhance the stability of hexons and protect the phage from releasing its DNA under extreme conditions [38]. There is evidence suggesting that pb10 exhibits an elongated density of binding to the hexon, with some preferred orientations, and has distinct binding sites in two types of hexons [26]. In contrast, our high-resolution structure indicated that the binding sites of pb10 were consistent in all hexons, likely due to minor conformational changes in the centers of the two types of hexons (Figure S6). We were unable to resolve the Ig-like domain of pb10 due to its flexibility (Figure 2C). Notably, pb10 may interact with host cells during infection [38]. Our findings indicate that pb10 provides structural stability and may facilitate initial host interactions in the early stages of infection.

### 2.3. Structure of the Connector Complex

**Figure 3:**
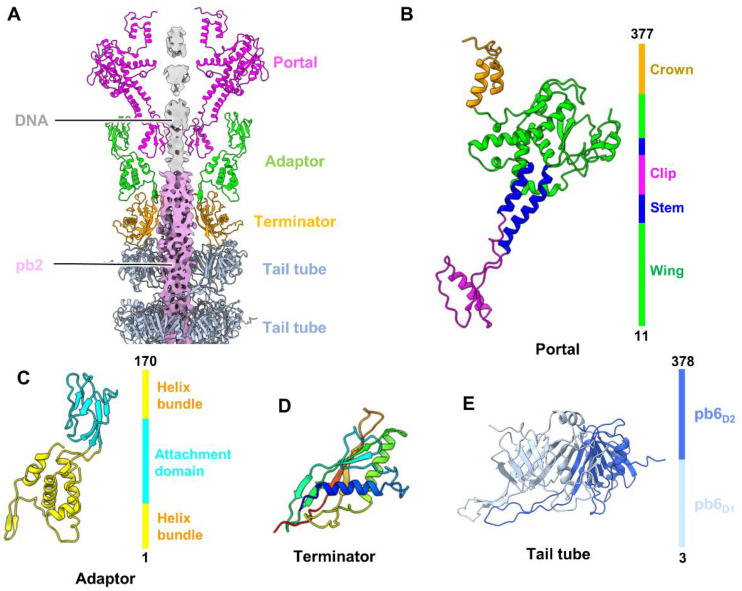
Structures of the connector complex and tail tube of mature T5. (**A**) Slab view of the ribbon models of the connector complex. The gray and pink in the middle are density maps of the DNA and TMP, respectively. (**B**,**C**) Ribbon models of portal protein pb7 (**B**) and adaptor protein p144 (**C**). (**D**) Ribbon model of tail terminator protein p142 shown in rainbow colors. (**E**) Ribbon model of the N-terminus of tail tube protein pb6. Model is colored according to domains. All the proteins are colored according to their domains.

The T5 connector complex, which connects the intricate tail to the icosahedral head, comprises three proteins: portal protein pb7, adaptor protein p144, and terminator protein p142 (Figure 1C and Figure 3A). The T5 portal consists of 12 copies of pb7, which replaces a penton at a unique vertex of the head and provides an entry and exit channel for DNA packaging and ejection. Based on our density map, we built an atomic model of pb7 from residues 11 to 377 of the total 403 residues (Figure S3A,B). According to the domain nomenclature of the portal protein, each pb7 monomer can be divided into four domains (Figure 3B): a crown domain (residues 337–377), a wing domain (residues 11–175, and 278–336), a stem domain (residues 176–200, and 263–277), and a clip domain (residues 201–262). The asymmetric reconstruction of the head–connector complex at a resolution of 10.5 Å revealed that the portal is in close proximity to a complex arrangement of double-stranded DNA (Figure S7). Imposing C12 symmetry on the portal by means of local reconstruction revealed that the wing domain’s positively charged residue, lys77, was anchored to the DNA fragment, clearly indicating the major and minor grooves of the double-stranded DNA (Figure S7). This DNA–portal interaction has previously been described for other phages, such as SU10 [8], P22 [39], and R4C [25], for which highly specific and stable interfaces are formed.

We built an atomic model of adaptor protein p144 comprising all 170 residues (Figure S3C). The adaptor is composed of 12 copies of p144, exhibiting structurally conserved conformation, as observed in many phages [40,41]. Each p144 monomer contains an attachment domain (residues 46–110) and a helix bundle domain (residues 1–45 and 111–170) (Figure 3C). The attachment domain is anchored to the outer surface of the portal, whereas the helix bundle domain is attached to the top of the tail terminator (Figure 3A). Notably, the loop (residues 14–20) of the helix bundle domain between two adjacent p144 monomers undergoes conformational changes to mediate the structural symmetry mismatch between the 12-fold symmetric adaptor and the 6-fold symmetric tail terminator p142 (Figure S8A,B).

We also built an atomic model of T5 terminator protein p142, including all 161 amino acid residues (Figure S3D). The tail terminator comprises six copies of protein p142. Each p142 contains two α-helices and a β-barrel structure facing the lumen of the tube (Figure 3D), which are homologous to the terminator proteins of other phages, such as Lambda (gpU) [23] and SPP1 (gp17) [42]. As observed for the T5 adaptor, substantial conformational changes occur in the C-terminus between two adjacent p142 monomers, which primarily mediate the structural symmetry mismatch between the six-fold symmetric tail terminator and the three-fold symmetric TTP (Figure S8C). Furthermore, the contact interface between the DNA and TMP was observed within the lumen of the adaptor-terminator channel (Figure 3A). In the SPP1 connector complex, the DNA’s terminal end is clamped by the tunnel loop of the portal, gp6, and is plugged at the stopper, gp16, to prevent its release [42]. In contrast, in the T5 connector, DNA may interact with multiple regions to prevent its release, including the negative electrostatic potential inside the connector channel and the presence of TMP in the tail tube, as previously proposed for phage DT57C [24] (Figure 3A and Figure S9).

The pairwise interfaces between the protein components of the T5 connector complex and the TTP are complementary in terms of electrostatic potential energy (Figure S10). In most myophages and siphophages studied so far, such as the Lambda [23], R4C [25], E217 [10], and Chi phages [43], the connector complex comprises four components: a portal, an adaptor, a stopper, and a tail terminator. The connector complex of some phages is surrounded by additional components. For example, in phages T4 [9], phiRSL1 [44], and XM1 [12], a ring collar encircles the connector complex, providing adsorption sites for the attachment of fibers or spikes, which are likely to mediate host–virus interactions. In the phage Milano, the collar comprises four stacked rings of the gp13 protein surrounding the connector complex. These rings contribute to signal transduction and the overall robustness of the phage particles against mechanical stress [45]. However, T5 and DT57C [24] lack a stopper in the connector complex, suggesting that the dodecameric adaptor may assume the stopper function, serving as a docking platform for the tail by binding to the tail terminator protein and ultimately completing the formation of the connector complex. Thus, T5 and DT57C may contain the minimal essential proteins required for the assembly of a functional connector complex.

### 2.4. Structure of the Tail Tube

The T5 tail tube comprises 40 stacked trimeric rings of TTP pb6, which form a right-handed helix with an axial rise of approximately 40 Å and a twist of approximately 39° for two adjacent rings (Figure 1A). The lumen of the tube is filled with TMP pb2 (Figure 1A). Each pb6 monomer contains three domains, two of these domains, pb6_D1_ and pb6_D2_, constitute the backbone of the tail tube, whereas the third is a protruding Ig-like domain [14]. We only built an atomic model for pb6_D1_ and pb6_D2_ from residues 3 to 378, which contain a β-barrel structure, including three α-helices and two long loops (Figure 3E and Figure S3E). However, the atomic model of the Ig-like domain of pb6 could not be built in our structure due to its flexibility. Ig-like domains at the tail tube periphery are very common in reported siphophages [23,24,43] and have been proposed to play accessory roles during infection, probably by binding to carbohydrates [46] or enhancing the biological activity of the tail tube [47]. Comparisons of the backbone of our TTP pb6 with those from X-ray crystallography [14] and from the previously reported T5 tip complex [18] reveal high levels of identity, with the exception of the long loop (Figure S11A). Additionally, structural superposition reveals that the whole X-ray structure of pb6 is well aligned with our low-resolution density map (Figure S11B). All these findings indicate that TTPs in T5 exhibit structural stability.

TTPs that form stacked hexameric rings exhibit common structural features across most myophages and siphophages [48], while the trimeric ring is unique to T5 and DT57C [24]. Despite the symmetry differences between the T5 TTP pb6 and other tubes, the pb6_D1_ and pb6_D2_ domains of the three pb6 monomers exhibit a pseudo-six-fold symmetric ring. The three pb6 monomers within the ring interact through the central β-barrel structure, with only long loops of one ring extending underneath to interact with the adjacent ring [14]. Structural comparisons of the first, middle, and last rings of the TTP pb6 reveal that only the long loop exhibits slight conformational changes (Figure S11C), indicating that TTPs may not play any direct role in infection, which is consistent with a previous structural study on TTP pb6 [14].

### 2.5. Structure of the Tail Tip Complex

**Figure 4:**
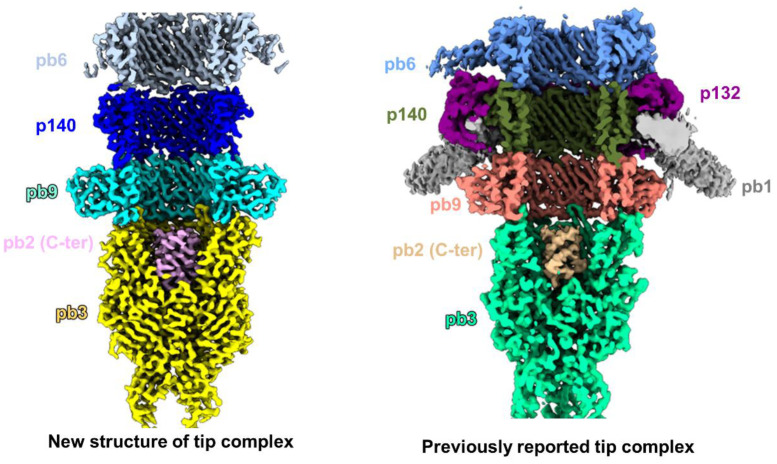
Density maps for the structure determined in this study and the previously reported (EMD-14869) tip complexes in mature T5. The newly determined structure lacks tail fiber pb1 and collar protein p132.

The distal end of the tail is occupied by the tail tip complex (Figure 1A). Based on our density maps, we built atomic models of proteins p140, pb9, and pb3, as well as the C-terminus of TMP pb2 within the lumen of the tail tube tip (Figure S3F–I). In comparison with a previously reported T5 tip complex structure [18], no density was observed for the three-fold side tail fiber pb1, or dodecameric collar protein p132 in our structure (Figure 4 and Figure S12). One study reported the absence of p132 in the fiberless T5 (referred to as T5hd1) through Western blot detection [28], and this finding is consistent with that obtained from our fiberless T5 in the present study. The tail tip structures of T5, including pb9, pb3, and the C-terminus of TMP pb2, are consistent with those of the aforementioned previously reported T5 tip complex [18], with the exception of p140. The flexible loops of p140 at the outer surface of the tip complex exhibit slight conformational changes due to the absence of p132 and pb1 adsorption (Figure S12B). A phage lacking tail fibers was constructed through selective breeding in the laboratory. Phage Lambda, which lacks side tail fibers, is used in many laboratories worldwide. The side tail fibers of Lambda facilitate its adsorption into the host cell receptor, although this may result in incorrect infection. Conversely, Lambdas lacking side tail fibers exhibit weak binding to the outer membrane but elevated infection accuracy [49]. Upon the removal of T5′s side tail fibers by mutation, the bacteriophage still forms plaques with high efficiency [50]. Furthermore, biochemical analyses in a previous study indicated that T5′s pb5 interacts solely with its purified host receptor, FhuA, to induce DNA release in vitro within a few seconds [29]. Thus, as with the fiberless Lambda, T5′s side tail fibers may not be essential for the host–virus interaction to trigger the DNA release. T5 lacking side tail fibers may employ a distinct strategy to initiate infection.

### 2.6. Conformation Changes in T5 from Mature to Urea-Treated Empty States

**Figure 5:**
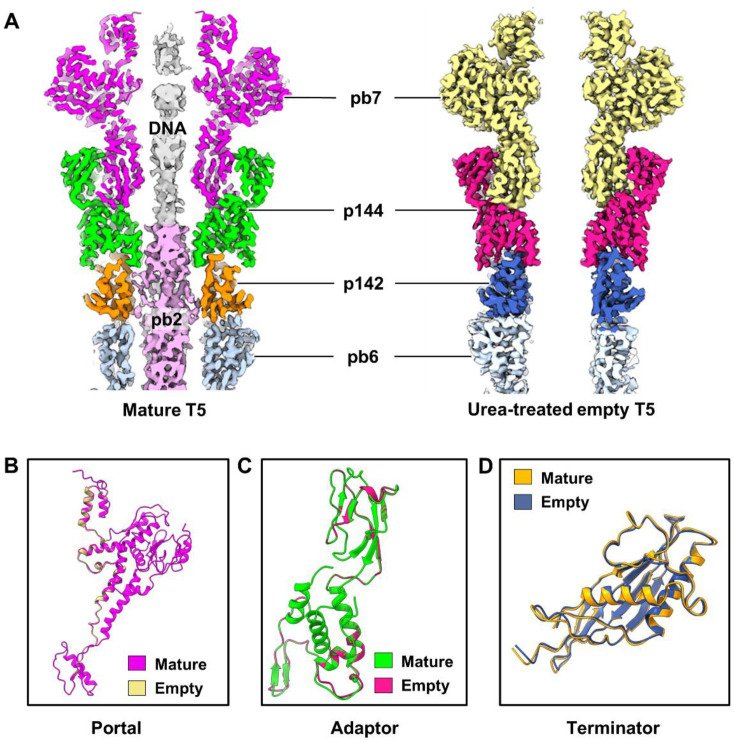
No apparent conformational change in the connector complex between mature T5 and urea-treated empty T5. (**A**) Slab views of the density maps of the connector complex in mature T5 (left) and urea-treated empty T5 (right). (**B**–**D**) Comparisons of the secondary structures of the portal protein (**B**), adaptor protein (**C**), and tail terminator protein (**D**) between mature T5 and urea-treated empty T5.

The release of the TMP and DNA in urea-treated T5 results in an empty tail and an empty head (Figure 1A,E). However, in both the mature and urea-treated states, the head remains unchanged due to the exceptional stability of the icosahedral shell. Structural comparisons of the portal, adaptor, and tail terminator proteins before and after DNA release indicated that no discernible conformational changes had occurred in these regions during infection (Figure 5). To date, obvious conformational changes following DNA release have only been described in the SPP1 connector complex [42]. However, most reported myophages and siphophages, such as DT57C [24], XM1 [12], and E217 [10], exhibit similarities to T5 in terms of the connector complex. In the mature state, the negative electrostatic potential inside the connector channel and TMP collectively prevent the release of DNA. In the DNA-ejected state, once the interaction of the tail tip complex or baseplate with the host cell induces the release of TMP [14,51,52], the large space of the connector complex enables the passage of DNA. Therefore, the connector complex may not be directly involved in the aforementioned processes. No significant differences were observed in the conformation of connector proteins before and after DNA release, and these proteins appear to be conserved across myophages and siphophages. Notably, the tip complex of the empty particle became detached for urea-treatment-induced reasons.

## 3. Discussion

In this study, we determined the structures of both mature T5 lacking side tail fibers and urea-treated empty T5 lacking a tail tip complex at near-atomic resolutions. High-resolution density maps enabled us to build accurate atomic models for MCP pb8 and decoration protein pb10, as well as for a tail tip complex lacking fiber pb1 and collar p132 in mature T5. Moreover, we resolved the structure of the connector complex in both the mature and urea-treated states, and this complex comprises portal protein pb7, adaptor protein p144, and terminator protein p142, which are the minimal essential proteins required to form a functional connector complex.

On the basis of the newly determined T5 structures, the aforementioned previously reported T5 tip complex [18] and a series of biochemical studies [27,28,29], we constructed complete models of mature and DNA-ejected T5. The models enabled us to explore the molecular mechanisms underlying the infection and DNA ejection of T5 lacking side tail fibers (Figure S13). It has been postulated that T5 does not require side tail fibers to orient itself vertically to the host cell surface for reversible adsorption in the early stages of infection [29,50]. Decoration protein pb10 in the T5 head may assist in infection to some extent [38]. RBP pb5 attaches vertically to the host receptor, FhuA (Figure S13A), triggering conformational changes in the tip complex (Figure S13B). The central fiber pb4 bends and brings the tail closer to the membrane, thereby opening the channel of the tip complex and anchoring the baseplate hub protein pb3 to the outer membrane [18]. Subsequently, the TMP is released, penetrating the host cell membrane and transmitting the signal to the phage capsid and DNA, thereby ultimately forming a channel that facilitates the entry of DNA into the host cell [18] (Figure S13C–E). Both the tip complex and the TMP play a pivotal role in the T5 infection process. Throughout this process, the connector complex remains unaltered, and TTPs serve solely as a framework, having no involvement in signal transduction or the expulsion of the TMP and DNA [14]. Our study provides a robust structural foundation to facilitate further investigations into the molecular mechanisms of infection and DNA ejection in other siphophages.

## 4. Materials and Methods

### 4.1. Production and Purification of T5

*E*. *coli* strain ATCC 11,303 was cultured in nutrient agar medium (8 g nutrient agar and 5 g NaCl per liter) for 24 h at 37 °C. Subsequently, T5 phages were incubated with the cells for 20 min at 37 °C. Afterward, the mixture was inoculated and amplified in 1 L of *E. coli* cells for 5 h at 30 °C. The cell lysate was separated by centrifugation at 9000× *g* for 30 min at 4 °C. Phage particles in the supernatant were precipitated by overnight incubation at 4 °C with 10% PEG8000 (Amresco, Solon, OH, USA) and 1 M NaCl (Aladdin, Shanghai, China), followed by centrifugation at 11,000× *g* for min at 4 °C. The precipitates were resuspended in buffer A (100 mM NaCl, 1 mM MgSO_4_, 1 mM CaCl_2_, and 10 mM Tris–HCl, pH 7.6) (Amresco, Solon, OH, USA), and the phages were purified by two rounds of CsCl gradient ultracentrifugation (Sigma, St. Louis, MO, USA). Initially, CsCl gradients (1.45, 1.56, and 1.70 g/mL CsCl) were used for purification and centrifuged at 135,000× *g* for 2 h at 4 °C. Subsequently, the infected phages were collected and re-centrifuged (1.56 g/mL CsCl) at 115,000× *g* for 8 h at 4 °C. The purified phages were then dialyzed overnight in buffer A. The final purified phages were stored in ice water for cryo-sampling.

Purified T5 phages were treated with 4 M urea (Amresco, Solon, OH, USA) for 2 h at 37 °C. Subsequently, samples of urea-treated empty T5 were dialyzed in buffer A to remove the urea. Finally, the dialyzed samples of empty T5 were concentrated by ultrafiltration for cryo-sample preparation.

### 4.2. Cryo-EM and Data Collection

An aliquot of 3 µL purified mature and urea-treated empty T5 was pipetted onto a Quantifoil R2/1 copper grid covered with a 3 nm thick layer of continuous carbon that had been glow-discharged for 15 s. The grids were loaded into an FEI Vitrobot (Thermo Fisher Scientific, Waltham, MA, USA) with 8 °C and 100% humidity, blotted for 4.0 s, and plunge-frozen in liquid ethane. The grids were subsequently stored in liquid nitrogen until data collection. Data were collected using an FEI Titan Krios G3i 300-kV electron microscope (Thermo Fisher Scientific, Waltham, MA, USA), equipped with a K3 summit direct electron detector (Gatan, Pleasanton, CA, USA) at 53,000× magnification, corresponding to a calibrated size of 1.36 Å/pixel. The accumulated dose of each movie was approximately 30 e^−^/Å^2^. In total, 3310 and 3897 movies of mature and urea-treated empty T5 particles were collected, respectively, and each movie stack comprised 32 image frames.

### 4.3. Data Acquisition and Icosahedral Reconstruction

The astigmatism and defocus values of each image were determined using GCTF [53] packaged in RELION 3.0 [54]. In total, 44,691 and 76,271 particle images of mature and urea-treated empty T5, respectively, were boxed out using the software EMAN 1.9 [55]. The icosahedral reconstruction of mature T5 was performed using our own programs [56] based on the common-line algorithm [57,58]. Subsequently, the three-fold region of the icosahedral head was reconstructed to a resolution of 3.4 Å by using the local reconstruction method [32,33].

### 4.4. Asymmetric Reconstruction of the Capsid–Connector Complex and Local Reconstruction of the Connector

The three-dimensional (3D) asymmetric structure of the head–connector complex of a mature T5 particle was reconstructed to a resolution of 10.5 Å by using the symmetry-mismatch reconstruction method [33,34], and the procedure can be described as follows: [1] Due to the high flexibility of the T5 tail, 44,691 particles containing the head and connector were manually selected. [2] For each particle image, the asymmetric orientation of each particle image was searched from the 60 equivalent orientations of its icosahedral orientation to reconstruct the head–connector complex as an initial model. [3] Using the newly determined orientations, a new asymmetric structure was reconstructed without imposing any symmetry. [4] Using the asymmetric structure from step [3] as the initial model, we repeated steps [2,3] in 12 rounds to improve the reconstruction resolution until most angles (~95%) remained unchanged. Subsequently, the resolutions of the connector complex and portal structures were refined to 4.8 Å and 3.2 Å by imposing 3-fold and 12-fold symmetry, respectively. The same method was then used to determine the structures of the entire head–connector complex and the connector complex of urea-treated empty T5 at resolutions of 7.5 Å and 4.2 Å, respectively.

### 4.5. Local Reconstruction of the Tail Tube and Tail Tip Complex

Local reconstruction of the tail tube and tail tip complex was performed using the RELION 3.0 software [35]. Specifically, 233,786 TTP particles were manually selected with a box of 360 × 360 Å by using the EMAN Helix software [59]. Using the previously reported tube structure of T5 as an initial model [14], we performed two-dimensional (2D) classification and 3D reconstruction by using the RELION 3.0 software [35]. A total of 199,483 particle images were selected for auto-refinement and 3D reconstruction by imposing three-fold symmetry. Finally, the resolution of the TTP was improved to a resolution of 3.6 Å.

To exclude non-tip or irrelevant structures, 60,595 tail tip complex particles with a box of 360 × 360 Å were manually selected for 2D classification. Subsequently, 45,023 particles were selected from the 2D classification to perform 3D classification by imposing three-fold symmetry, based on a random cylinder density as an initial model. This process yielded four low-resolution structures with varying center positions. Based on the first type, *Z*-axis translation was performed for each of the three types to correct the center and re-box tail tip images. A total of 30,589 particle images were selected for 3D refinement, achieving a resolution of 3.9 Å, by imposing three-fold symmetry.

### 4.6. Atomic Model Building and Refinement

Based on the cryo-EM density maps of mature T5, we manually built models for MCP pb8, the N-terminus of decoration protein pb10, portal protein pb7, adaptor protein p144, tail terminator protein p142, the N-terminus of TTP pb6, distal tail protein pb9, p140, baseplate hub protein pb3, and the C-terminus of TMP pb2 by using the COOT 0.9.4 software [60]. Based on density maps of urea-treated empty T5, we built models for portal protein pb7, adaptor protein p144, and tail terminator protein p142. All the models were refined using the real-space refinement method, implemented in Phenix [61]. The refinement and validation statistics are presented in Table S1.

## Data Availability

The cryo-EM electron density maps for the 3-fold region of the T5 capsid, the portal complex of mature T5, the connector complex of mature T5, the tail tube of mature T5, the tail tip complex of mature T5, the portal complex of urea-treated empty T5, and the connector complex of urea-treated empty T5 have been deposited in the EM Data Bank under the accession codes EMD-60511, EMD-60672, EMD-60675, EMD-60712, EMD-60750, EMD-60695, and EMD-60689, respectively. The atomic coordinates for the 3-fold region of the T5 capsid, the portal complex of mature T5, the connector complex of mature T5, the tail tube of mature T5, the tail tip complex of mature T5, the portal complex of urea-treated empty T5, and the connector complex of urea-treated empty T5 have been deposited in the Protein Data Bank under the accession codes 8ZVI, 9ILP, 9ILV, 9INY, 9IOZ, 9IMV, and 9IMH, respectively.

## Supplementary Materials

The following supporting information can be downloaded at: https://www.mdpi.com/article/10.3390/ijms25158479/s1.
