# Supplementary material for: Structures of Mature and Urea-Treated Empty Bacteriophage T5: Insights into Siphophage Infection and DNA Ejection
Source: Int J Mol Sci. 2024 Aug 3;25(15):8479. doi: 10.3390/ijms25158479 (PMC11313276; doi:10.3390/ijms25158479)
Supplement: Supplementary file 1 [file ijms-25-08479-s001.zip › ijms-3108018-supplementary.pdf]

Supplemental Information

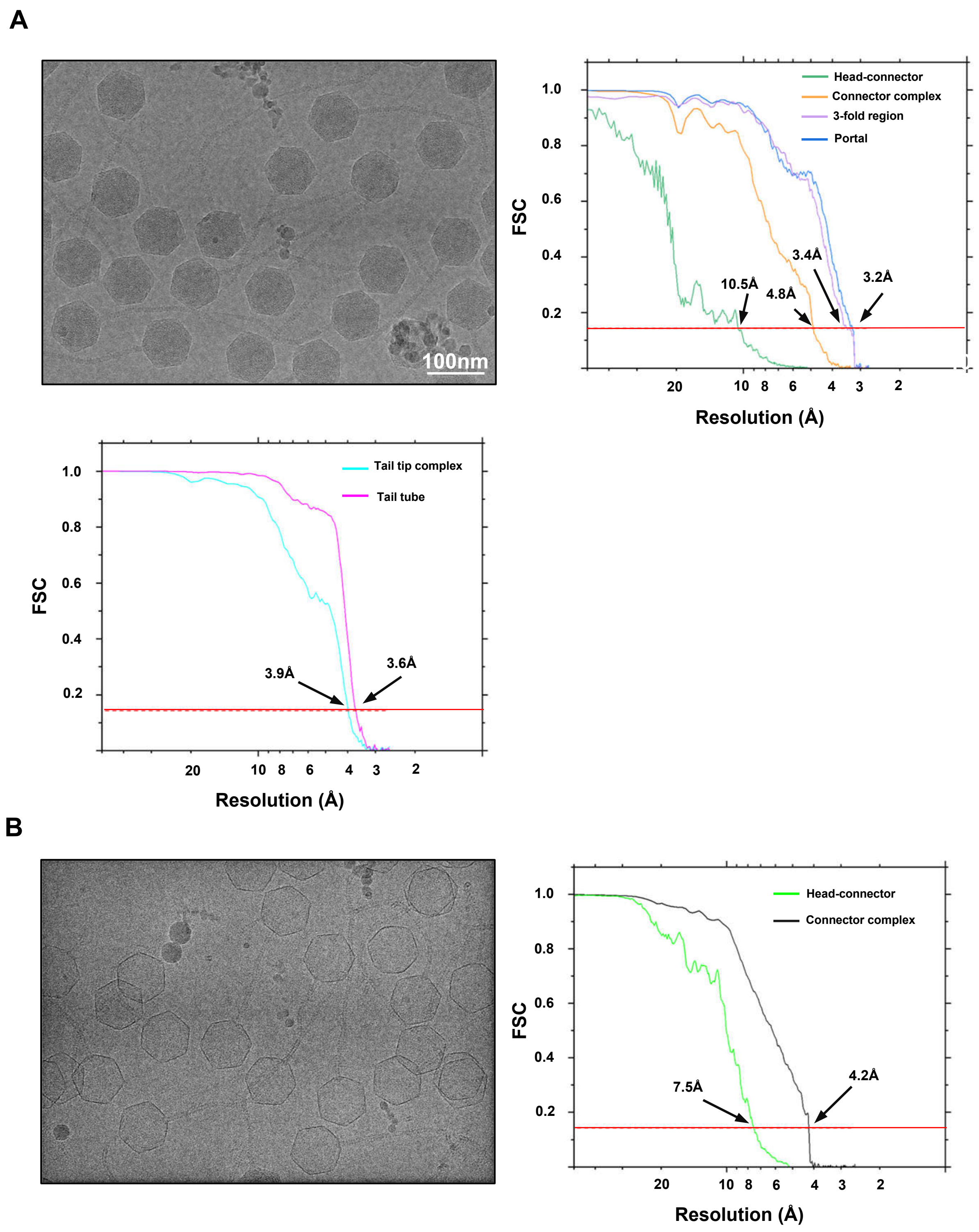

Figure S1. Cryo-EM images and Fourier shell correlation curves of mature T5 and urea-treated empty T5. (A) Representative cryo-EM images of mature T5 and estimated structural resolutions of the head-connector structure (green line), connector complex (orange line), 3-fold region of the icosahedral head (purple line), portal (blue line), tail tip complex (cyan line), and tail tube (magenta line) in mature T5. **(B)** Representative cryo-EM images of urea-treated empty T5 and estimated structural resolutions of the head-connector structure (lime line) and connector complex (black line) in urea-treated empty T5.

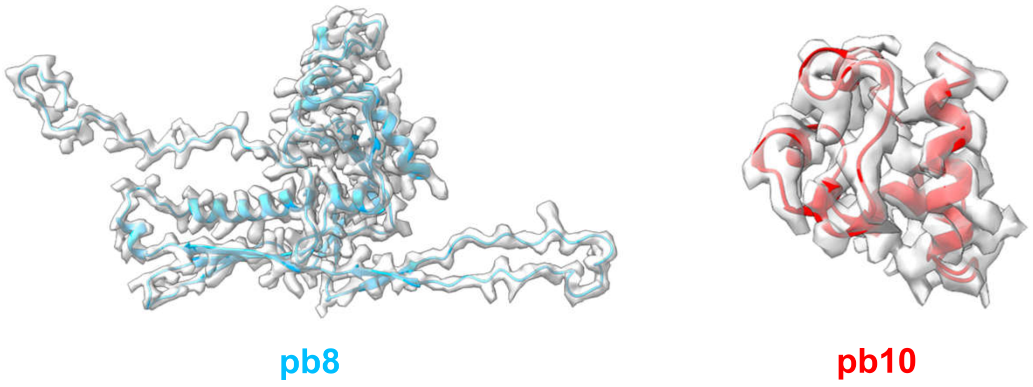

Figure S2. Ribbon models of mature T5's major capsid protein pb8 (left) and decoration protein pb10 (right) superimposed on their density maps.

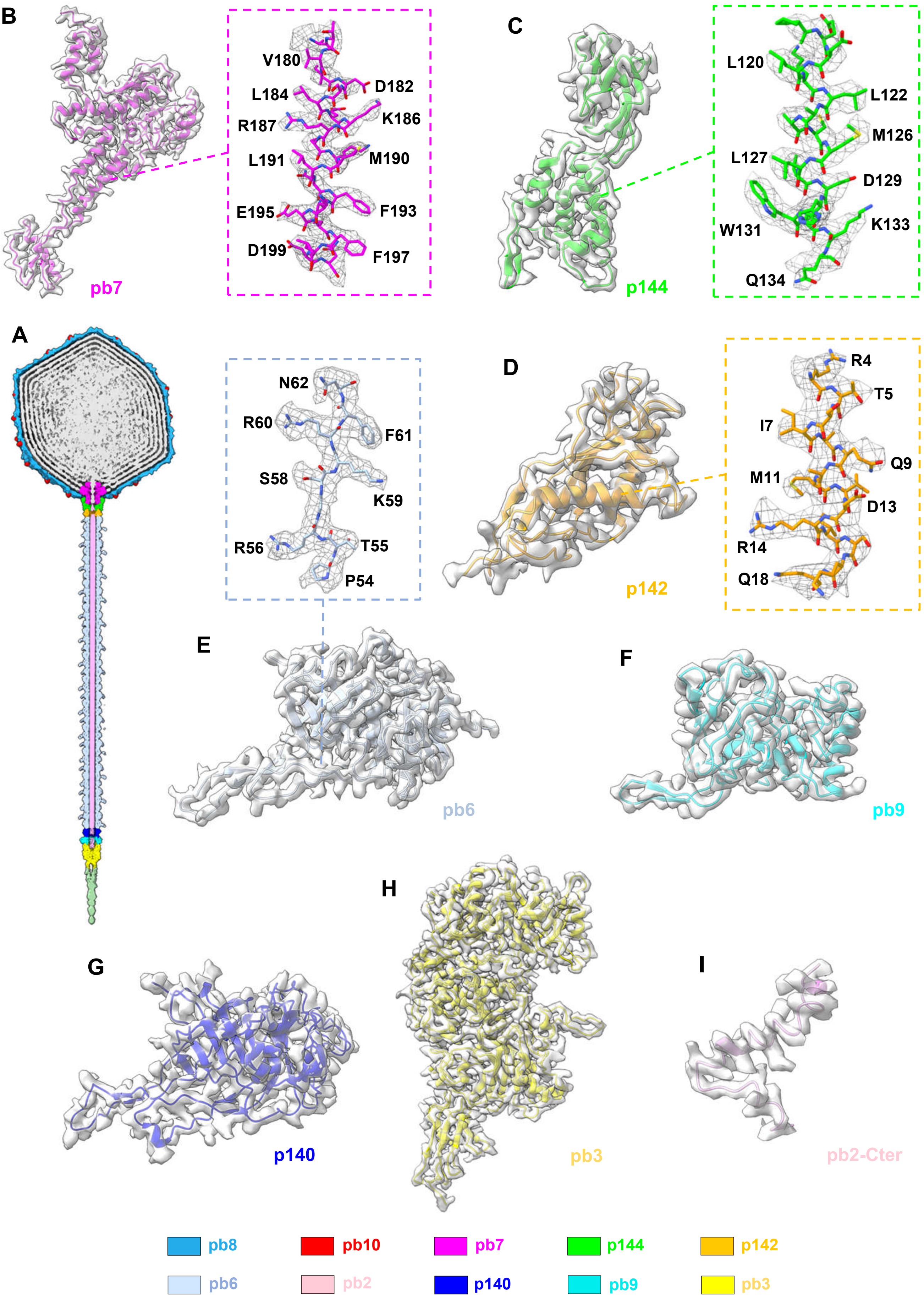

Figure S3. Quality of the cryo-EM density maps and atomic models of the connector complex and tail in mature T5. (A) Side view of the entire asymmetric structure of mature T5. **(B-I)** Ribbon models of the connector-tail proteins, and zoomed-in views of density maps (mesh) superimposed on their atomic models (sticks). The color code is identical to that in Fig. 1A.

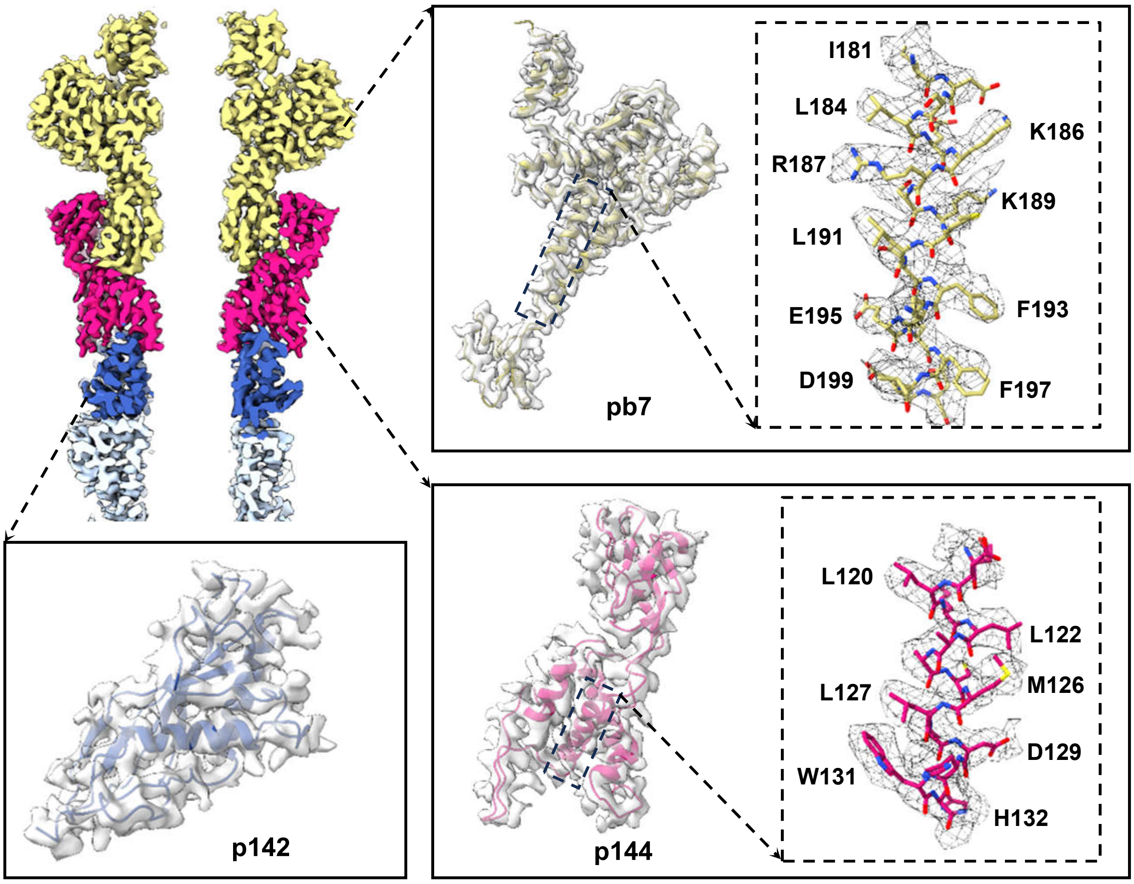

Figure S4. Quality of the cryo-EM density maps of the connector complex in urea-treated empty T5. Zoomed-in views of density maps (mesh) are superimposed on their atomic models (sticks). The color code is identical to that in Fig. 5A.

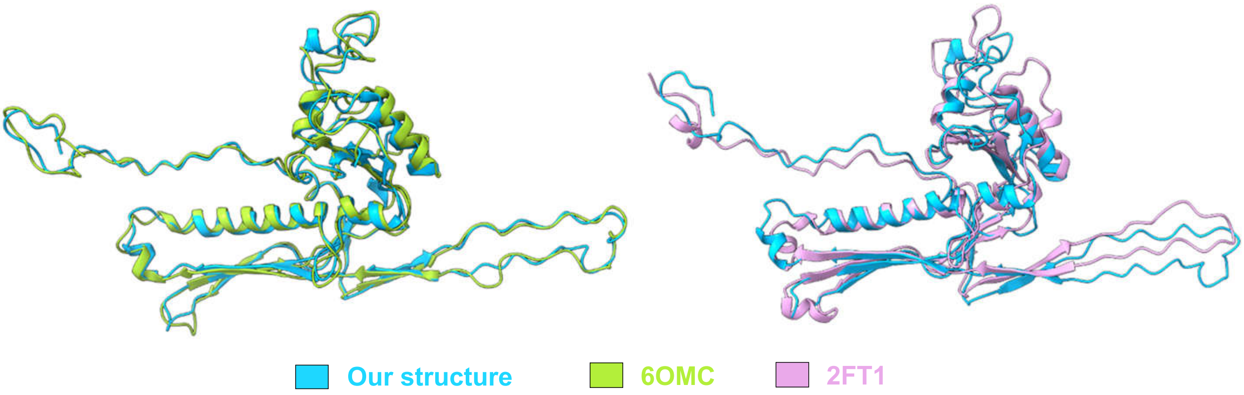

Figure S5. Comparisons of secondary structures of the T5 MCP monomer in this study with the previously reported T5 MCP monomer (left, PDB ID: 6OMC) and HK97 MCP monomer (right, PDB ID: 2FT1).

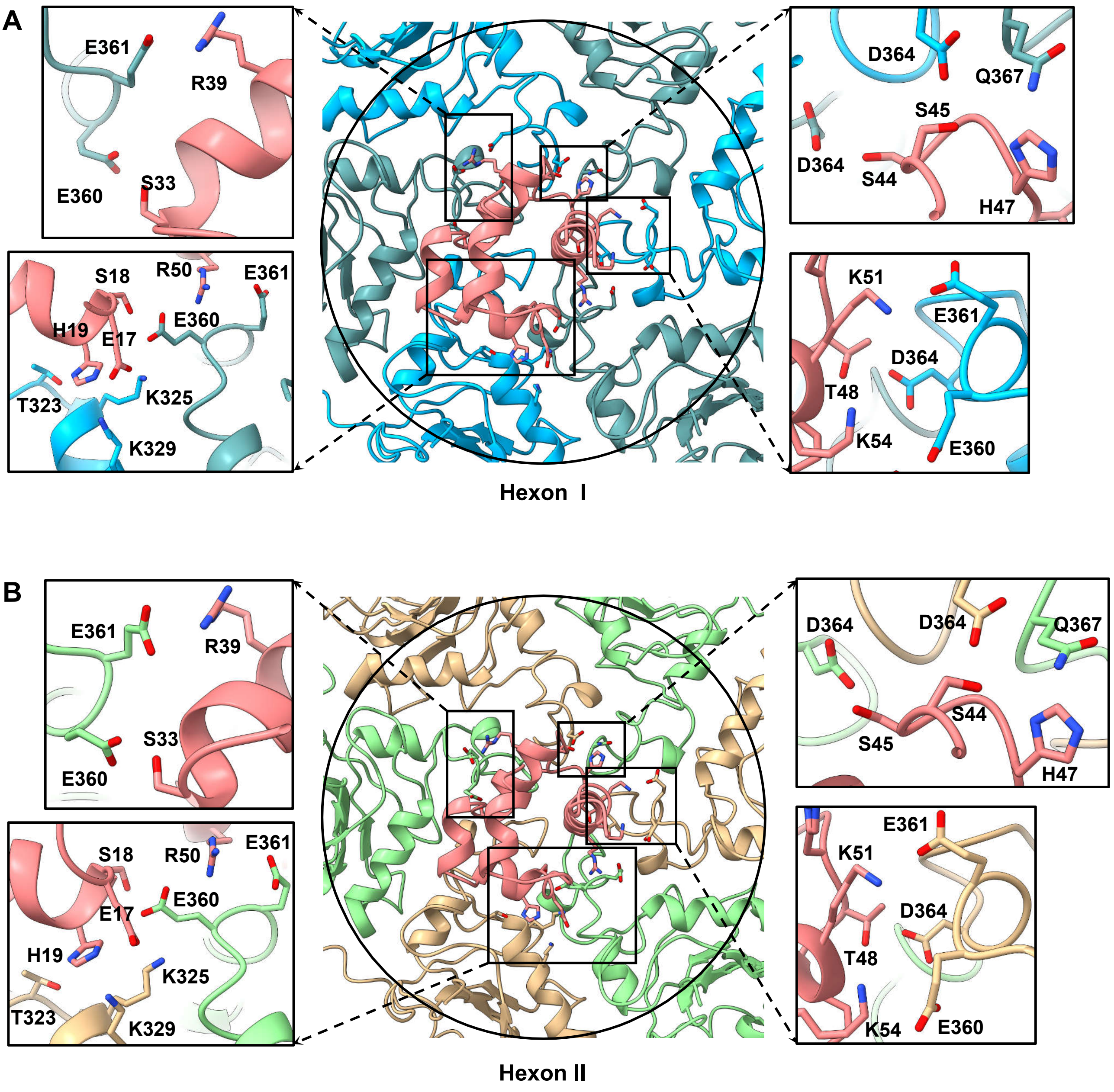

Figure S6. Identical interactions between the pb10-hexon I and pb10-hexon II interfaces in the icosahedral head. Six pb8 monomers in hexons I and II are alternatively colored in cadet blue and sky blue, green and yellow, respectively, pb10 is colored in carnation.

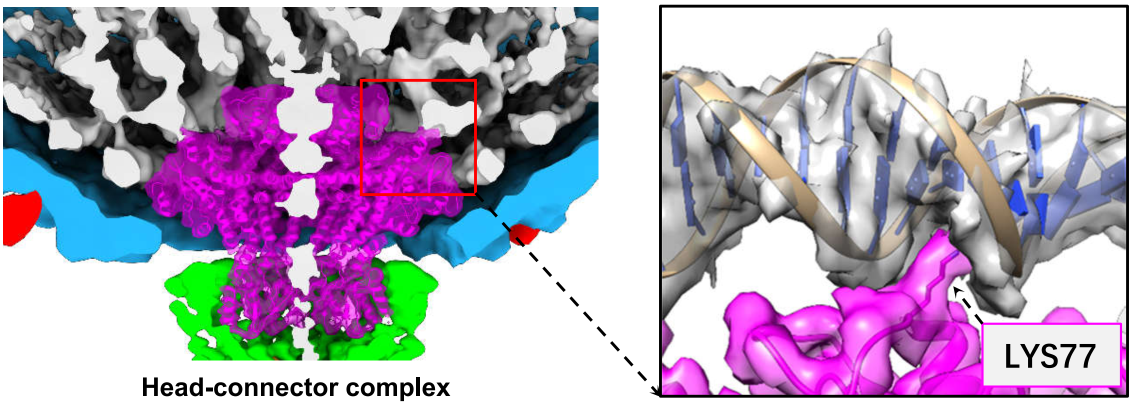

Figure S7. Interactions between the portal and DNA in mature T5. Zoomed-in view shows a close interaction between residue lys77 of the portal's wing and the DNA fragment.

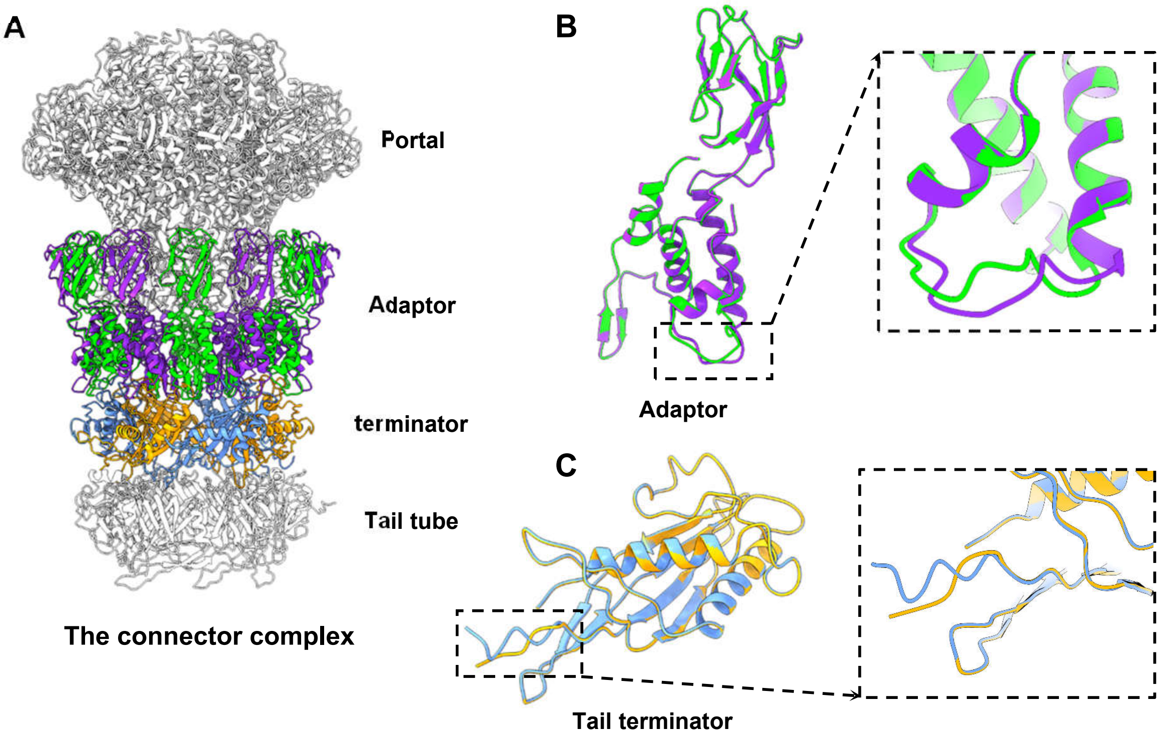

Figure S8. Interactions between the connector complex and tail tube of mature T5. (A) Side view of the atomic models of the connector complex and tail tube. The adaptor and the tail terminator are alternatively colored in green and violet, blue and orange, respectively, and the portal and the tail tube are colored in gray. **(B and C)** Structural comparisons of two adjacent monomers in the adaptor (B) and tail terminator (C), and zoomed-in views depicting conformational differences.

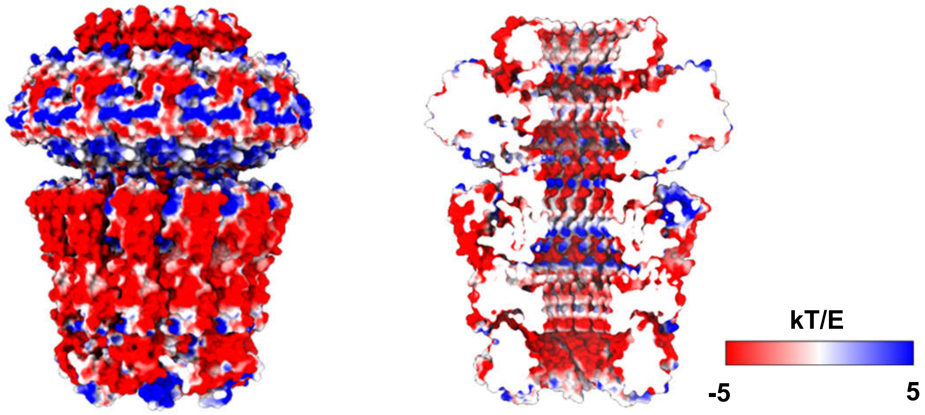

Figure S9. Side (left) and cut-open (right) views of electrostatic potential surfaces of the outer and inner surfaces of the connector complex in mature T5. The electrostatic potential scale is shown in the color bar.

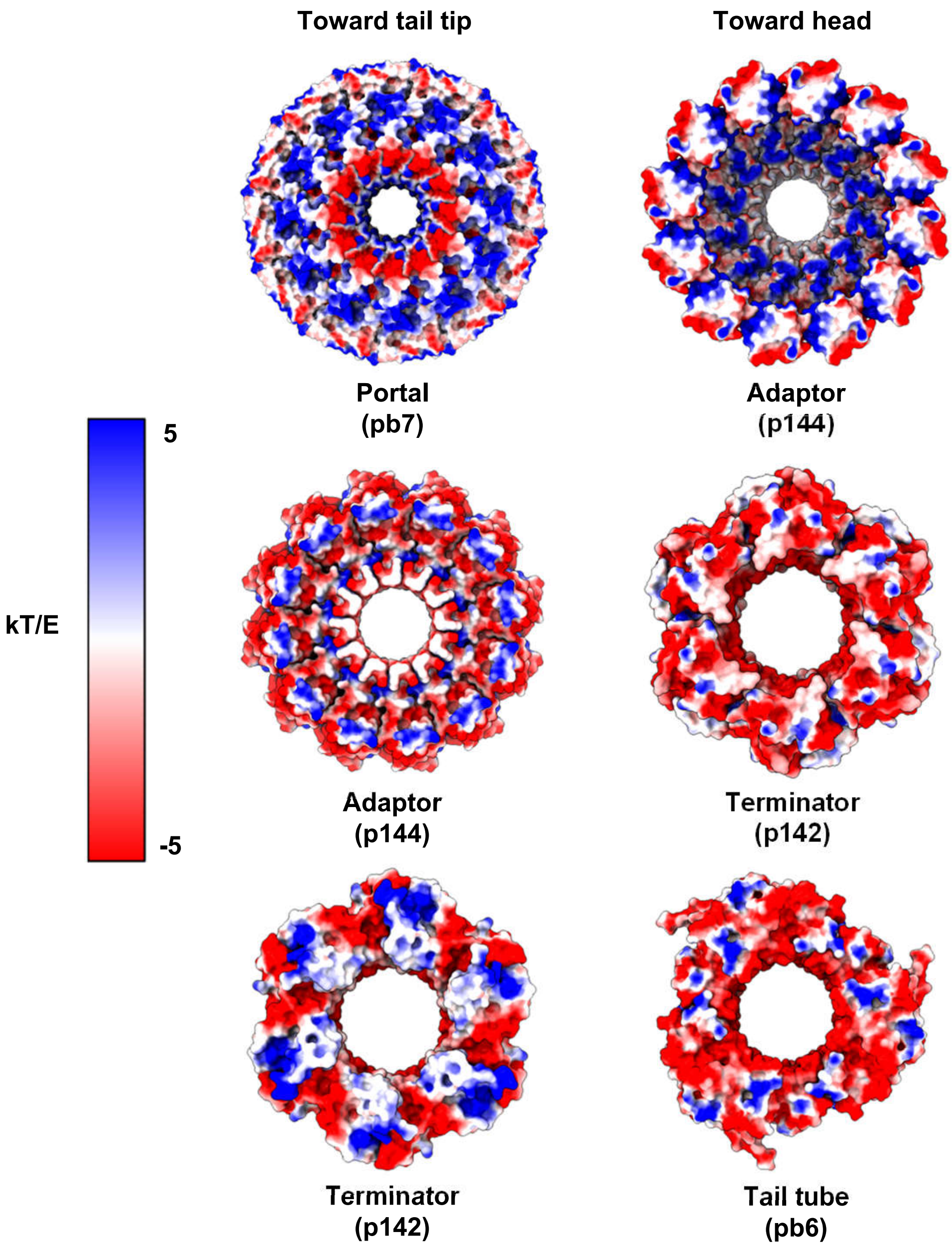

Figure S10. Electrostatic potential surfaces of the interacting regions of two adjacent protein components in mature T5. The left columns are oriented toward the tail tip, whereas the right columns are oriented toward the head. The electrostatic potential scale is shown in the color bar.

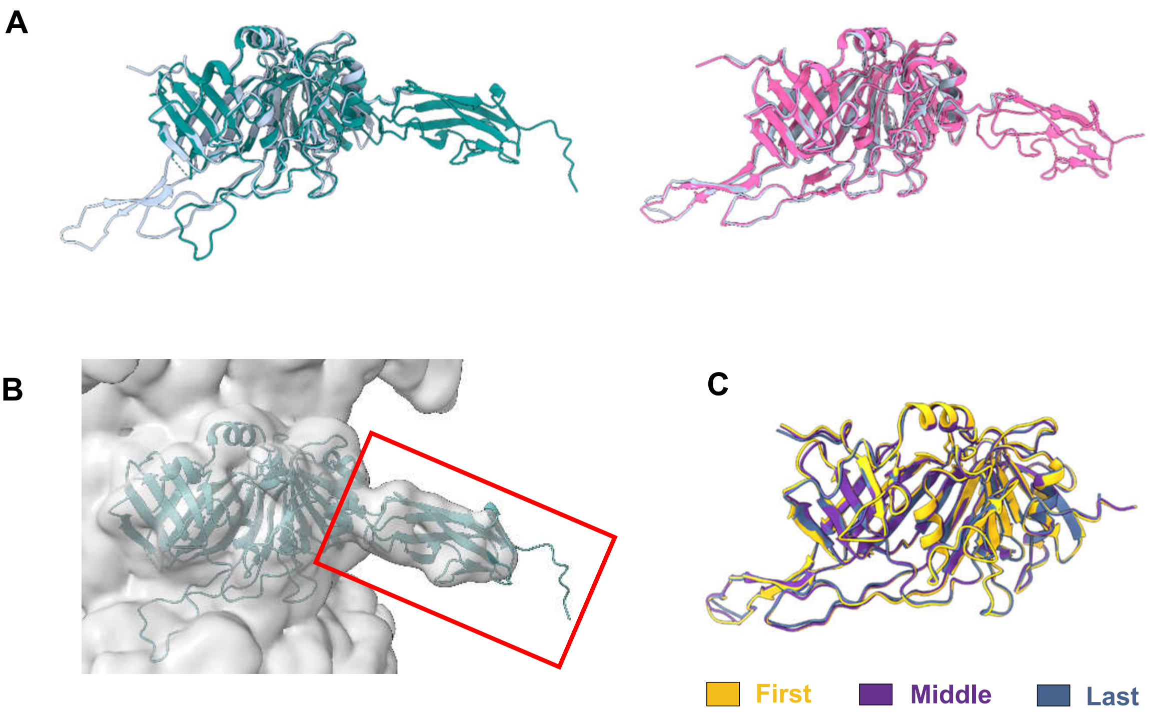

Figure S11. Structure of the tail tube of mature T5. (A) Structural comparisons of the TTP pb6 in this study (light blue) with the X-ray structure of pb6 (PDB ID: 5NGJ, cadet blue) and the pb6 structure (PDB ID: 7QG9, pink) from the previously reported tip complex. **(B)** Ribbon models of X-ray structure of pb6 (PDB ID: 5NGJ, cadet blue) superimposed on our low-resolution density map of TTP. **(C)** Structural comparison of the atomic models of TTP pb6 among the first, middle, and last rings in this study.

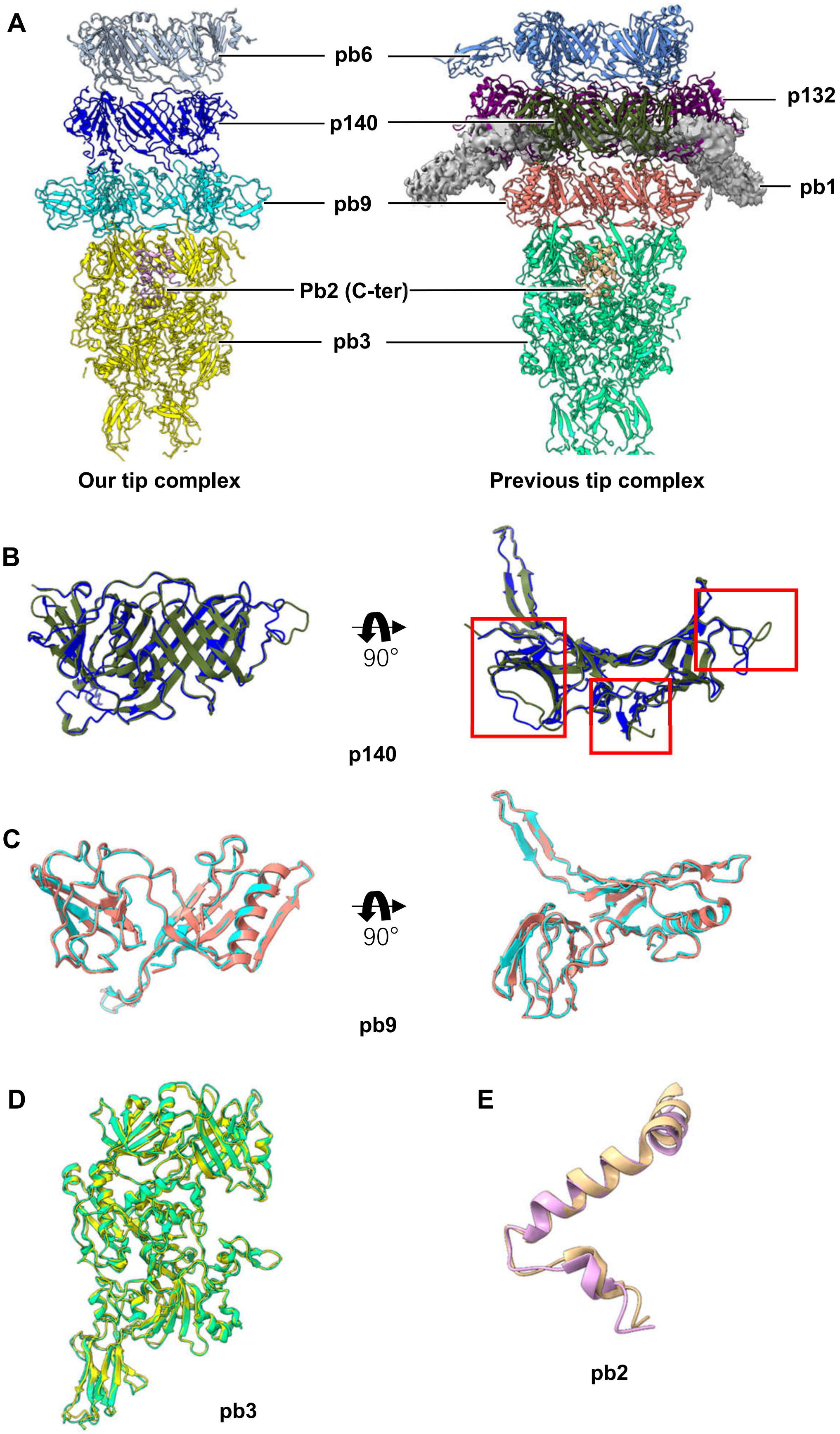

Figure S12. Structural comparison of proteins in different T5 tail tip complexes. (A) Side views of the atomic models of the newly determined (left) and previously reported (right, PDB ID: 7QG9) tip complexes. **(B-E)** Comparisons of the secondary structures of p140 (B), pb9 (C), pb3 (D), and pb2 (E) between the determined and previously reported tip complexes, depicting minor conformational changes except for p140.

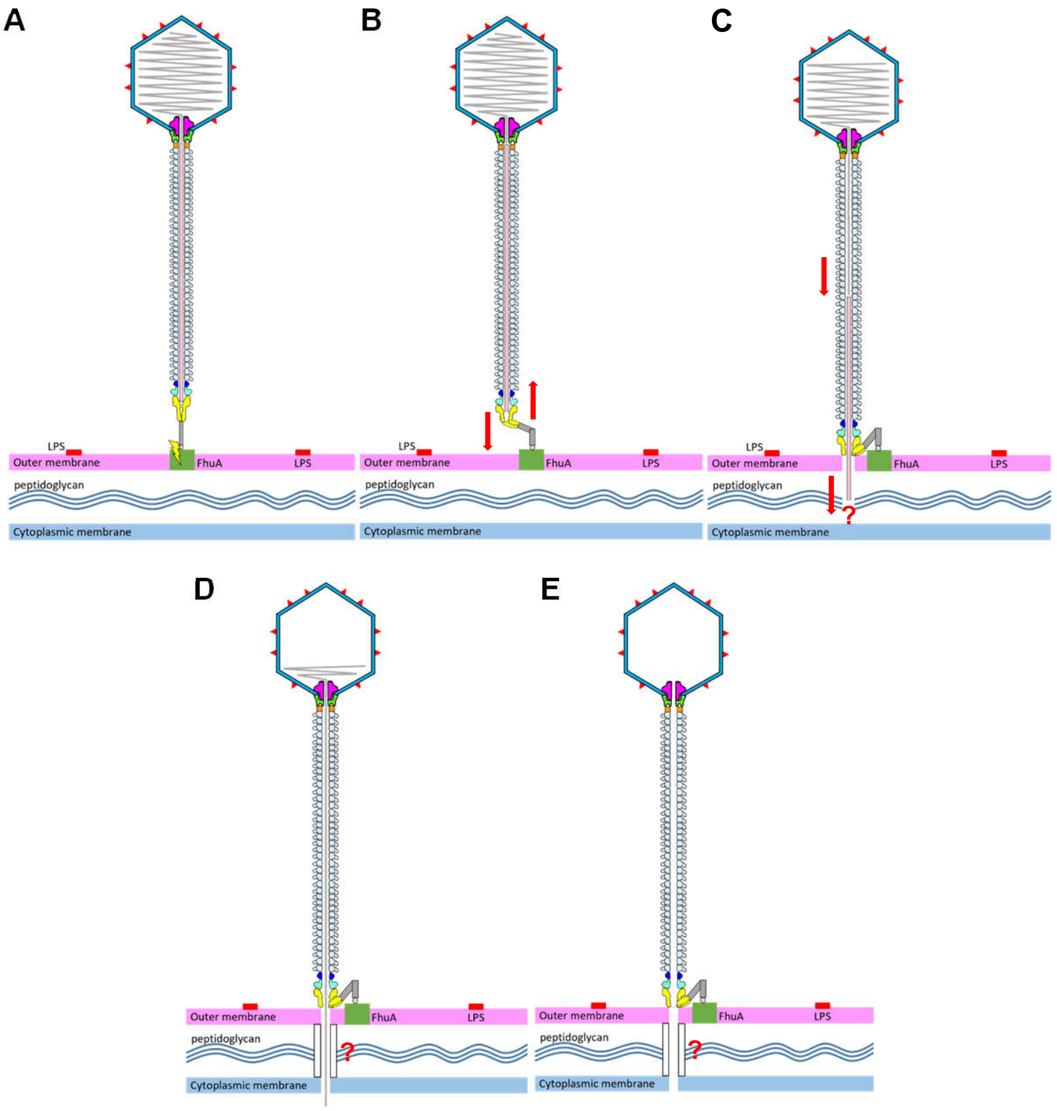

Figure S13. Schematic diagram of T5 infection and DNA-ejection pathway. (A) The RBP pb5 binds to the host receptor FhuA. **(B)** The central fiber pb4 bends and brings the tail closer to the outer membrane. **(C)** The TMP is subsequently released. **(D)** The DNA exits from head to the host cell. **(E)** Empty T5. The color coding is identical to that in Fig. 1.

Table S1 Refinement and model statistics.

**Data collection**												
Electron microscopy	Titan Krios G3i											
Pixel size (Å)	1.36											
Defocus range	1.6 to 2.2um											
Total movie-mode micrographs	Mature phage T5								Urea-treated empty T5			
	3,310								3,897			
**Symmetry-mismatch reconstruction**												
	Head-connector in mature phage T5								Head-connector in urea-treated empty T5			
Total particles	44,691								76,271			
Resolution(Å)	10.5								7.5			
**Local reconstruction**												
	Mature phage T5									Urea-treated empty T5		
	Head		Portal		Connector complex	Tail tube	Tail tip complex			Connector complex		
Total particles	36,710		18,398		18,398	199,483	30,589			76,271		
Resolution (Å)	3.4		3.2		4.8	3.6	3.9			4.2		
EMDB ID	60511		60672		60675	60712	60750			60689		
**Atomic models refinement/statistics (phenix.real_space_refine）**												
	Mature phage T5									Urea-treated empty T5		
	Head		Connector complex			Tail tube	Tail tip			Connector complex		
Protein	pb8	pb10	pb7	p144	p142	pb6	p140	pb9	pb3	pb7	p144	p142
PDB ID	8ZVI		9ILP		9ILV	9INY	9IOZ			9IMV		9IMH
Model Resolution in Refinement (Å)	3.4		3.2		4.8	3.6	3.9			4.2		
Total Residues	297	66	367	170	161	376	293	204	840	367	170	161
CC (model to map fit)	0.8059		0.8171		0.7617	0.7872	0.8274			0.843		
Ramachandran most favorable (%)	92.18		91.92		83.76	92.25	86.15			91.32		
Ramachandran additionally allowed (%)	7.47		6.89		13.92	6.95	12.26			7.74		
Ramachandran disallowed (%)	0.36		1.19		2.32	0.8	1.59			0.93		
